# Nigeria’s race to zero COVID-19 cases: True disease burden or testing failure?

**DOI:** 10.7189/jogh.11.03094

**Published:** 2021-08-07

**Authors:** Ahmad Ibrahim Al-Mustapha, Abubakar Ahmed Tijani, Muftau Oyewo, Ahmed Ibrahim, Nusirat Elelu, Oluwaseun Adeolu Ogundijo, Emmanuel Awosanya, Annamari Heikinheimo, Victoria Olusola Adetunji

**Affiliations:** 1 Department of Veterinary Services, Kwara State Ministry of Agriculture and Rural Development, Ilorin, Kwara State, Nigeria; 2 Department of Veterinary Public Health and Preventive Medicine, Faculty of Veterinary medicine, University of Ibadan, Oyo State, Nigeria; 3 Faculty of Pharmaceutical Sciences, Université de Tours, Tours, France; 4 Nigeria Field Epidemiology and Laboratory Training program, Asokoro, Abuja, Nigeria; 5 African Center for Disease Prevention and Control, Abuja, Nigeria; 6 Department of Veterinary Public Health and Preventive Medicine, Faculty of Veterinary Medicine, University of Ilorin, Kwara state, Nigeria; 7 Department of Food Safety and Environmental Health, Faculty of Veterinary Medicine, University of Helsinki, Finland

The 2019 coronavirus disease (COVID-19), caused by a novel member of the beta coronaviruses, severe acute respiratory syndrome coronavirus 2 (SARS-CoV-2), has caused a global public health hazard [1]. Since Nigeria’s index case on the 27 February 2020, the country has tested 1 818 957 persons, recorded 163 195 confirmed cases and 2058 deaths as of 31 March 2021 [2]. The positivity rate among the tested population was 9.0% and the case fatality rate was 1.3%. In April 2020, the Nigerian Government, through the Nigeria Center for Disease Control and Prevention (NCDC), activated its national Incident Control Center (ICC), the Surveillance and Outbreak Response Management System (SORMAS), and the mobile strengthening epidemic response system (mSers) amongst others for national coordination, surveillance and reporting of COVID-19 cases in the country [3].

Nigeria’s health care system is sub-optimal and plagued by a plethora of challenges as seen in many other low- and middle-income countries [4]. Nigeria’s current health expenditure is 3.75% of its gross domestic product (GDP) [5]. With a Physician to population ratio of 4:10 000, Nigeria has one of the worst physicians to population ratio in the world [6]. The pandemic has further stretched the health care workforce, created panic in health facilities and made obvious the dilapidated situation of the country’s health infrastructures [3,4]. Another major constraint to the success of Nigeria’s COVID-19 control strategy was the high human poverty index (HPI). In Nigeria, an estimated 83 million people (40%) live below the poverty line [7], thus making COVID-19 a disease of hunger [8].

An effective COVID-19 testing system is critical to understanding the pandemic’s burden and spatio-temporal spread across the globe. This should enable the prompt identification of infected persons, determining populations most at risk, improve prognosis, and assist with contact tracing [3]. However, Nigeria’s COVID-19 testing system and capacity are sub-optimal with close to only 2 000 000 people tested out of the estimated 210 000 000 population. Despite the low testing capacity, an even lower incidence of COVID-19 cases is being reported daily (**Figure 1**). Hence, this viewpoint asks the million-dollar question: Have we flattened the curve? And if yes, how soon do we get to zero cases? Furthermore, we evaluated Nigeria’s COVID-19 testing capacity 14 months after the identification of patient zero.

**Figure 1:**
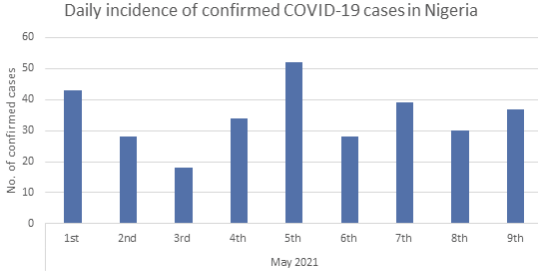
Daily incidence of confirmed COVID-19 cases in Nigeria as of 9 May 2021 [2].

## COVID-19 TESTING AND LABORATORY CAPACITY

A reliable, fast, and efficient testing system is crucial to identifying cases and curbing the spread of COVID-19 especially in densely populated countries such as Nigeria (210 million people). Prior to the COVID-19 pandemic, Nigeria’s laboratory networks and diagnostic capacities were poor. This was evident in the number of existing laboratories by January 2020, as only 6 BSL-3 laboratories with capacity to test for SARS-CoV2 existed in Nigeria. These facilities were mainly conducting quantitative polymerase chain reaction (RT-qPCR) and other confirmatory diagnostic tests for important endemic and sporadic outbreaks such as Lassa fever, Yellow fever and Cholera [3,4]. These laboratories soon became overwhelmed with the increasing COVID-19 incidence in Nigeria. As of April 2020, Nigeria could only test 2500 samples per day. To cater to the rising disease burden, NCDC leveraged on two existing platforms (HIV molecular testing laboratories as well as the GeneXpert *M. tuberculosis* testing equipment) and repurposed these machines to test for SARS-CoV-2. As of April 5, 2021, with increased commitment from the Government there now exists 124 COVID-19 testing centers in Nigeria. Of these, 76 are public owned COVID-19 testing facilities in the NCDC network, while 41 are fee-paying private COVID-19 testing laboratories and 7 corporate laboratories spread across Nigeria [2].

However, despite the expansion of the laboratory network across the country, only about 1.8 million samples have been tested across all platforms in Nigeria (open PCR, GeneXpert, and RDT kits) over 14 months (March 2020 – April 2021). This represents only 0.8% of Nigeria’s population [9]. **Table 1** puts into perspective, Nigeria’s COVID-19 tests done in comparison with few selected countries. The average daily COVID-19 tests conducted rose from 2500 samples in April 2020 to 4000 samples by December 2020 and 5250 samples by April 2021. Currently, Nigeria has only tested 8.5 persons per thousand population. Hence, we opine that this extremely low testing system could be responsible for the low daily COVID-19 incidence in Nigeria.

**Table 1:** Cumulative COVID-19 tests conducted by selected countries as of 31 March 2021

Country	Population [10]	Total humans tested [9]	Tested per thousand [9]	Daily average [9]
Nigeria	210 134 412	1 803 177	8.5	5250
Ethiopia	117 790 090	2 301 145	19.8	8200
Morocco	37 245 243	5 443 945	147.5	11 300
South Africa	59 881 455	9 803 871	165.3	27 000
UAE	9 981 853	37 354 626	3776.9	220 000
United Kingdom	68 155 831	124 147 198	1783.5	1 216 000

## RAPID DIAGNOSTIC TESTS

Although RT-PCR is the gold standard for diagnosing COVID-19, antigen-based rapid diagnostic tests (RDTs) was granted emergency use authorization by the World Health Organization (WHO) in September 2020 [11]. RDTs are cheap, fast, require less technical expertise and could be more sustainable in LMICs such as Nigeria. Consequently, Nigeria validated it during the screening of youth corps before their admission into the compulsory one-year national service [12]. Ige et al. reported that although COVID-19 RDTs have lower sensitivity and specificity, they have proven useful in scaling up Nigeria’s COVID-19 testing system especially in schools, congregations, and hard-to-reach areas [13]. However, it is yet to be determined if the low sensitivity of RDTs contributed to the lower COVID-19 incidence in Nigeria. COVID-19 testing using RDTs should be conducted by qualified health care personnel while adhering strictly to COVID-19 protocols to avoid technical errors and further community spread of the SARS-CoV-2 [13].

**Figure:**
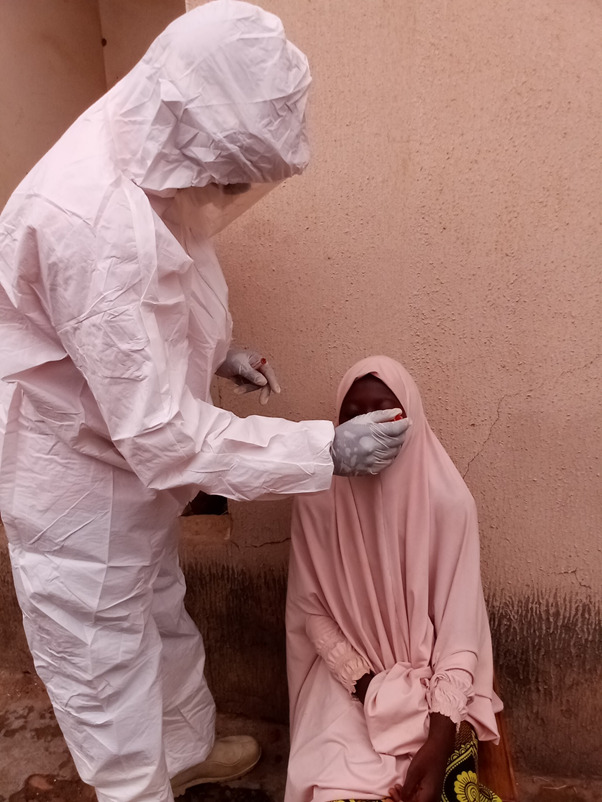
Photo: From the collection of Al-Mustapha Ahmad Ibrahim (used with permission).

## THE NEED TO REVIEW THE NATIONAL COVID-19 TESTING PLAN

To successfully navigate the nation out of this pandemic, the true burden of the COVID-19 pandemic must be determined especially in the race to zero cases. This can be achieved by increasing Nigeria’s COVID-19 laboratory testing capacity across the nation. The current testing framework and strategies are ineffective and must be improved so that increased testing can be done to capture at least 5% of Nigeria’s population by December 2021. Furthermore, we propose “risk-adjusted mass screening” of Nigerians. This is in line with the target of the Africa Center for Disease Control and prevention (Africa CDC) initiative “partnership to accelerate COVID-19 testing” (PACT) which aims to conduct at least 80 000 tests per million population [14]. Some African countries have used this testing system to determine the spatio-temporal spread and the true burden of COVID-19 in their countries. Countries such as South Africa (9 803 871), Morocco (5 443 945) etc. have started the risk-based mass screening of their citizens [14]. In some of these countries, the local production of their nucleic acid extraction kits and laboratory reagents has assisted them in ramping up the number of tests conducted. PACT, Coalition against COVID-19 (CA-COVID), and other corporate organizations have assisted the Presidential Task Force (PTF) to increase testing, contact tracing, and treatment of all cases, which are cardinal components in reaching zero cases [14].

The need for community engagement towards any successful public health intervention cannot be overemphasized. Hence, to fully curtail the spread of the SARS-CoV-2, there is the need to employ community engagement strategies that will encourage adoption of all recommended safety measures through rigorous public education and community enforcement in order to prevent further spread of the virus within the country. This should be combined with rigorous contact tracing, improved testing strategy and mass vaccination with effective vaccines. The government must improve public trust in its handling of the pandemic through mass advocacy and public health education. Public trust in government is a crucial factor in vaccine acceptance, a cardinal instrument in curbing the spread of the SARS-CoV-2 and returning to normalcy. Therefore, it remains paramount for the government to sensitize and persuade its citizenry to “take responsibility” by strictly adhering to the infection prevention and control measures (use of face mask, frequent hand washing, physical distancing) and accepting the COVID-19 vaccine.

## CONCLUSION

Although government’s preparedness and response efforts towards the pandemic have been above average, more needs to be done especially in the area of testing. Nigeria has neither flattened the curve nor heading towards zero COVID-19 cases. The low daily disease incidence is mostly a function of a very low testing rate. There is a need to quickly roll out COVID-19 vaccination to cover 70% of the population so as to achieve herd immunity. Furthermore, a transparent, holistic, and participatory approach by the government is critical to combating the COVID-19 pandemic in Nigeria and rebuilding stronger health systems towards adjusting to a “new normal”.
